# Iron-dependent essential genes in *Salmonella* Typhimurium

**DOI:** 10.1186/s12864-018-4986-1

**Published:** 2018-08-14

**Authors:** Sardar Karash, Young Min Kwon

**Affiliations:** 1 Cell and Molecular Biology Program, University of Arkansas, Fayetteville, AR USA; 2 Department of Biology, College of Education, Salahaddin University, Erbil, Kurdistan Iraq; 3 Department of Poultry Science, University of Arkansas, Fayetteville, AR 72701 USA

**Keywords:** *Salmonella* Typhimurium, Essential genes, Iron-restriction, Reactive oxygen species, Antibiotic targets

## Abstract

**Background:**

The molecular mechanisms underlying bacterial cell death due to stresses or bactericidal antibiotics are complex and remain puzzling. Due to the current crisis of antibiotic resistance, development of effective antibiotics is urgently required. Previously, it has been shown that iron is required for effective killing of bacterial cells by numerous bactericidal antibiotics.

**Results:**

We investigated the death or growth inhibition of *S.* Typhimurium under iron-restricted conditions, following disruption of essential genes, by transposon mutagenesis using transposon sequencing (Tn-seq). Our high-resolution Tn-seq analysis revealed that transposon mutants of *S.* Typhimurium with insertions in essential genes escaped immediate killing or growth inhibition under iron-restricted conditions for approximately one-third of all previously known essential genes. Based on this result, we classified all essential genes into two categories, iron-dependent essential genes, for which the insertion mutants can grow slowly if iron is restricted, and iron-independent essential genes, for which the mutants become nonviable regardless of iron concentration. The iron-dependency of the iron-dependent essential genes was further validated by the fact that the relative abundance of these essential gene mutants increased further with more severe iron restrictions. Our unexpected observation can be explained well by the common killing mechanisms of bactericidal antibiotics via production of reactive oxygen species (ROS). In this model, iron restriction would inhibit production of ROS, leading to reduced killing activity following blocking of essential gene functions. Interestingly, the targets of most antibiotics currently in use clinically are iron-dependent essential genes.

**Conclusions:**

Our result suggests that targeting iron-independent essential genes may be a better strategy for future antibiotic development, because blocking their essential gene functions would lead to immediate cell death regardless of the iron concentration. This work expands our knowledge on the role of iron to a broad range of essential functions and pathways, providing novel insights for development of more effective antibiotics.

**Electronic supplementary material:**

The online version of this article (10.1186/s12864-018-4986-1) contains supplementary material, which is available to authorized users.

## Background

Essential genes encode the proteins that are essentially required for cell viability or growth. These genes have been exploited as pivotal targets for antibacterial drugs, because blocking their proteins cause cell impairment and ultimately growth inhibition or death of bacterial cells. Thus, nearly all antibiotics in clinical use target these essential pathways. However, for many natural antibiotics, the molecular targets remain unknown [1] and even if the target is known, in case of bactericidal antibiotics, the cellular events that follow in response to disruption of essential pathways leading to bacterial cell death have remained to be explored.

Numerous studies have shown the role of reactive oxygen species (ROS) in cell death for eukaryotes as well as prokaryotes. In eukaryotes, apoptosis and necroptosis are associated with ROS [2, 3]. Ferroptosis is an iron-dependent nonapoptotic form of oxidative cell death in mammalian cancer cells. These cells die as a result of ROS accumulation and the death can be prevented via iron chelators [4]. In bacteria, contribution of ROS to cell death due to bactericidal antibiotics is elucidated by recent studies. Kohanski et al. [5] proposed that bactericidal antibiotics, regardless of their molecular targets, induce production of ROS that contributes to cell death, and also demonstrated that the death process can be mitigated via iron chelators. This model asserts that upon antibiotic-target interactions, consecutive specific intracellular events induce ROS formation, specifically hydroxyl radical, via Fenton reaction through the process that involves TCA cycle-NADH depletion and destabilization of Fe-S clusters [5, 6]. Furthermore, it was shown that ROS generation elevates in bacterial cells by the attack of competitor bacteria or P1vir phage via type VI secretion system [7]. In addition, mammalian peptidoglycan recognition protein-induced bacterial killing requires ROS and the lethality of this protein can be inhibited via an iron chelator [8]. Immune cells also produce ROS to kill bacterial pathogens [9]. However, despite these numerous evidences on the role of ROS in bacterial cell death, it is unknown if this role of ROS can be extended to all death processes in bacterial cells, and if not, what the scope of the essential genes is for which ROS production contributes to cellular death when they are inactivated or their protein functions are blocked.

A bacterium can possess a few hundred essential genes that are indispensable for maintaining cell viability. Empirically, essential genes are defined as the genes that when inactivated lead to loss of cell viability. In *E. coli* Keio collection, single-gene deletions were made for all known open reading frames, excluding 302 genes which could not tolerate disruptions and these 302 genes were considered essential [10, 11]. On the other hand, random genome-wide transposon mutagenesis coupled with next generation sequencing (Tn-seq) is a powerful method to identify essential genes [12]. Tn-seq experiments have shown that there are 353 essential genes in *Salmonella* Typhimurium SL326 [13]; 461 in *Mycobacterium tuberculosis* H37Rv [14]; and 227 in *Streptococcus pyogenes* [15]. Recently, a synthetic strain *Mycoplasma mycoides* JCVI-syn3.0 was created based on 473 essential genes [16]. In a recent study, Clustered Regularly Interspaced Short Palindromic Repeats Interference (CRISPRi) was employed for phenotypic analysis of 289 essential genes in *Bacillus subtilis* that were identified by Tn-seq, and confirmed that approximately 94% of the putative essential genes were genuine essential genes [17].

Nearly all studies on defining essential genomes in bacteria have been conducted using stress-free nutrient-rich media for the given bacterial species under the assumption that a minimum set of the core essential genes would be best revealed under such “optimal” growth conditions. In the current study, on the contrary, we analyzed our Tn-seq data to determine essential genes in *S.* Typhimurium under the stress conditions created by restricting iron concentrations using iron chelator 2,2`-Dipyridyl (Dip) ranging from 0 to 400 μM. Our initial focus was to identify conditionally essential genes required for fitness under iron-restricted conditions. However, we unexpectedly found that a significant portion of the genes that are categorized as essential genes in LB media (no Dip) were rendered non-essential under iron-restricted conditions. Furthermore, the relative abundance of the transposon mutants with insertions in those essential genes increased with the increasing severity of iron restrictions. We reason that this finding has significant implications for the current efforts to overcome the crisis in public health due to increasing antibiotic resistance, and may provide valuable insights for future direction for the development of new antibiotics with inherent mechanisms for reduced chance of developing drug resistance. Therefore, in this study we focus our analysis on the essential genes of *S*. Typhimurium under iron-replete and iron-restricted conditions, and discuss the implications of our discovery. The result on conditionally essential genes under iron-restricted conditions will be reported elsewhere.

## Results

### Library selection and initial Tn-seq analysis

We constructed two genome-saturating Tn5 transposon libraries (Library–A and –AB) in which 92.6% of all ORFs had insertions (Additional file 1: Table S1). To track the relative abundance of mutants in the libraries in response to iron restriction, appropriate library was inoculated into LB media supplemented with iron chelator 2,2`-Dipyridyl (Dip) at final concentrations of 0 (controls: LB-II and LB-III), 100 (Dip100), 150 (Dip150), 250 (Dip250-I and Dip250-II), or 400 μM (Dip400) (Additional file 2: Figure S1). The cultures were grown until the bacterial growth reached mid-log phase. Since the growth rate of *S.* Typhimurium was reduced due to Dip treatment in a concentration-dependent manner (Additional file 2: Figures S2 and S3), the cultures were incubated for different lengths of time to reach mid-log phase (Additional file 1: Table S1). We also included Library-A itself (LB-I) as an additional reference for Tn-seq analysis (Additional file 2: Figure S1). For Library-AB, we obtained 273 million (M) sequence reads of Tn5 genomic junctions on the chromosome of *S*. Typhimurium for all conditions combined, and 185 M sequence reads were mapped to the completed genome of *S*. Typhimurium 14,028 (Additional file 1: Table S2).

The accurate genome-mapping based on long Tn5 genomic junctions and high number of read counts allowed us to define essentiality and conditional essentiality of the genes with high precision. Our initial goal in this study was to elucidate the conditionally essential genes that are required for fitness under different levels of iron restriction. During the data analysis, however, we found that the read counts corresponding to the mutants in numerous known essential genes increased significantly and consistently under iron-restricted conditions. This observation was in contrary to the currently accepted working definition of essential genes that cannot tolerate disruptions. It required further detailed analysis before we could accept this interesting, yet unexpected finding. Therefore, we have conducted a systematic analysis for the essential genes, and comparatively analyzed the results between iron-replete and iron-restricted conditions.

### Essential genome of *S*. Typhimurium in iron-replete and iron-restricted conditions

We used a rigorous analytical pipeline for essential gene identification as outlined in Additional file 2: Figure S4. As a result, we identified 336 essential genes that are required for aerobic growth of *S*. Typhimurium 14028 in LB broth and on LB agar plate (Additional file 1: Table S3). We compared these essential genes in *S*. Typhimurium 14028 to those in *S*. Typhimurium SL3261, which was previously identified by TraDIS [13]. Interestingly, out of the 336 genes in our essential gene list, 265 (80%) orthologous genes in *S*. Typhimurium 14028 were also essential in *S.* Typhimurium SL3261 (Additional file 1: Table S4). Further, KEGG pathway analysis recognized 306 out of the 336 genes, which were categorized into 23 essential pathways (Additional file 2: Figure S5).

**Fig. 1:**
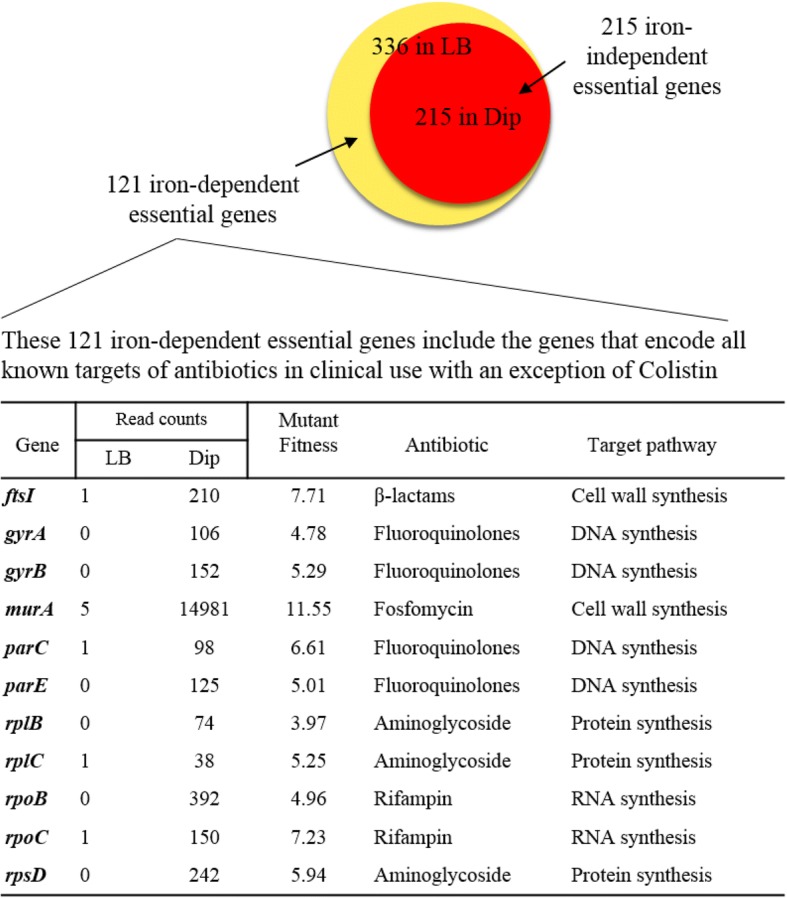
Iron-independent vs. iron-dependent essential genes in *S.* Typhimurium. Essential genes of *S.* Typhimurium were identified using Tn-seq method in LB medum (iron-replete) and LB medium supplemented with 2,2`-Dipyridyl (Dip; iron-restricted). Among all 336 essential genes identified, 215 genes were essential under both conditions (iron-independent essential genes), while 121 genes were essential only under iron-replete condition (iron-dependent essential genes). The iron-dependent essential genes included 11 genes encoding well known antibiotic targets. The read counts from LB-III (LB) and Dip400 (Dip) were used to calculate mutant fitness as expressed in Log_2_ fold change

We also used the same analytical pipeline to identify the essential genes under iron-restricted conditions. Surprisingly, the number of essential genes under iron-restricted conditions decreased to 215 genes, which indicated that 121 genes (36%) of the 336 essential genes in LB media were considered non-essential under iron-restriction conditions (Additional file 1: Table S5). The number of reads corresponding to the insertions in these 121 genes significantly increased under iron-restricted conditions: the average read counts per gene for the 121 genes were 4.4 in LB-III, whereas this elevated to 67.9 in Dip400 (Additional file 1: Table S6). This is a clear evidence that the mutants of the 121 genes escaped immediate killing or growth arrest, and were able to multiply slowly under iron-restricted conditions. In another word, the 121 genes that were essential in the absence of chelator (LB medium; iron-replete condition) became non-essential in the media supplemented with the iron chelator (iron-restricted conditions) (Fig. 1, Additional file 1: Tables S6 and S7). Therefore, based on this finding, we classified the 336 essential genes in *S*. Typhimuirum into two categories, 121 iron-dependent and 215 iron-independent essential genes. The mutants of iron-dependent essential genes can escape immediate killing and grow slowly under iron-restricted conditions, whereas the mutants of iron-independent essential gene die or could not multiply in both iron-replete (LB medium) and iron-restricted conditions.

### Fitness of the mutants of iron-dependent essential genes

**Fig. 2:**
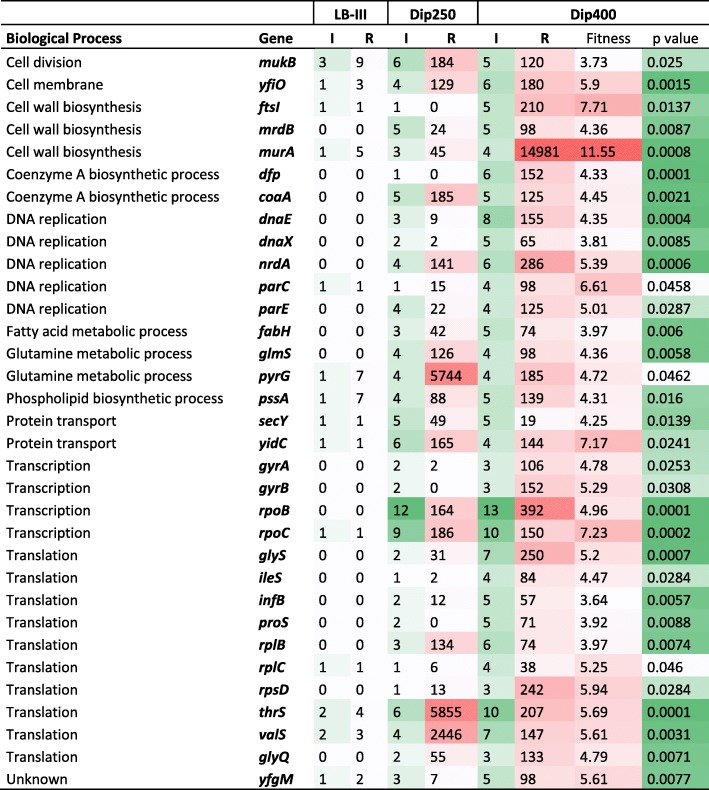
Iron-dependent essential genes have increased number of insertion sites and read counts under iron-restricted conditions. A genome-saturating library of *S.* Typhimurium Tn5 mutants was selected in LB medium (iron-replete; LB-III) and LB medium supplemented with Dip (iron-restricted; Dip 250 and Dip400). The resulting mutant pools were subjected to Tn-seq analysis to identify essential genes. Mutant fitness in Dip400 increased significantly in comparison to LB-III for the 33 genes shown here (*P* < 0.05). I and R indicate the number of unique insertion sites and total read counts, respectively. Mutant fitness is expressed in Log_2_ fold change of the sequence read counts in Dip400 in comparison to LB-III

We next measured the changes in mutant fitness for the 121 iron-dependent essential genes in the presence of high concentrations of Dip using Dip250-I, Dip250-II and Dip400 Tn-seq data as the outputs in comparison to LB-III as the input. Strikingly, the mutant fitness of 97 out of 121 genes (78%) increased numerically (fitness expressed in Log_2_ fold change in read counts) in Dip400, and 8 other genes showed increased mutant fitness in Dip250-I, Dip250-II or both. Further statistical analysis revealed that the mutants of the 33 essential genes showed significant increase in fitness in the presence of Dip, including *gyrA*, *gyrB*, and *ileS* (*p* value < 0.05; Fig. 2). For these 33 genes, the mutants showed at least Log_2_ fold change in read counts ≥3.64 in Dip400 in comparison to LB-III. Interestingly, *gyrA*, *gyrB* and *ileS* were not in the list of 121 iron-dependent essential genes (considered as iron-independent essential genes), whereas fitness of these mutants increased significantly by iron restriction. In the case of *murA*, the read counts increased from 5 (LB-III) to 14,981 (Dip400), exhibiting highest increase in abundance (Fig. 2).

### Iron-dependent essential genes are not condition-specific essential genes

It is well known that the essential genes are operationally defined depending on the variations in the specific optimal growth conditions used for the experiment. We then asked if our discovery could be considered as a general extension of the concept to iron-restricted conditions. To answer this question, we closely examined our Tn-seq data that were generated for other stress conditions such as H_2_O_2_ [18] and H_2_O_2_ coupled with Dip (unpublished). However, we could not find any similar patterns that a significant portion of the essential genes in LB medium became nonessential under stress conditions. We also examined other studies on identification of essential genes in other bacteria, including *S.* Typhimurium SL326 [13], *Mycobacterium tuberculosis* H37Rv [14] and *Mycoplasma mycoides* JCVI-syn3.0 [16] as well as *E. coli* [10]. Nearly all orthologous essential genes in *S*. Typhimurium, particularly the 33 essential genes described above, are also considered essential in rich media for those bacterial species mentioned above (Additional file 1: Table S8). Further, Lee et al. [19] used Tn-seq to identify essential genes in *Pseudomonas aeruginosa* under 6 different conditions, and found that the 352 genes are general essential genes important in all 6 different media, while 199 essential genes were condition-specific. The condition-specific essential genes constitute 11–23% essential genes depending on the growth medium. It is important to note that all these 6 media support the growth of *P. aeruginosa* well, and thus are not considered as stress conditions. In contrast, in our study 36% of the essential genes in LB medium became nonessential under iron-restricted stress conditions, and there were no condition-specific essential genes for iron-restricted condition (Fig. 1). Therefore, we argue that these 121 genes should be still considered as essential genes according to the current definition of essential genes, instead of condition-specific essential genes in LB medium. In other words, these 121 genes are general essential genes, but became nonessential under this particular condition (iron-restricted condition) due to the unusual role iron plays in broad range of death processes in *S.* Typhimurium following inactivation of the essential genes.

### Validation of the Tn-seq results

We next asked whether the increase in the number of unique insertion sites and read counts for the 121 iron-dependent essential genes under iron-restricted conditions was due to a bias in Tn-seq data analysis. We conducted the analysis for identification of essential genes in this study without data normalization. Typically, normalization of read counts is critical for reliable identification of conditionally essential genes for the mutant libraries before and after the selection. On the contrary, essential gene analysis is usually conducted without normalization. It is because the process is based on one Tn-seq profile from the particular optimal condition under which the essential genes are studied, and the information on insertion sites is critical for discovery of essential genes, while relative abundance of mutants is not considered. In this study, there was considerable variations in the total read count of Tn-seq profile across the mutant pools (Additional file 1: Table S2). Furthermore, when focused on the reads in ORFs only, the total read counts of LB-III was ~ 30 million (M) versus ~ 16 M in Dip400, excluding intergenic regions (Additional file 1: Table S9). Interestingly, the insertions in two genes, *STM14_2422* (*umuC*) and *STM14_2428*, consumed 8.7% of all reads in LB-III and 27% in Dip400. Consequently, excluding the reads from these two genes, an insertion in LB-III is expected to have 227 reads on average, while 100 reads for an insertion in Dip400 (Additional file 1: Table S9). This indicates that the bias in read counts was toward lower read counts for the same genes under iron-restricted conditions (Dip400) as compared to LB-III. Nevertheless, the read counts of the insertions in the 121 iron-dependent essential genes were higher in Dip400 as compared to LB-III. This is a strong evidence that the mutants in these 121 genes did indeed multiply slowly under iron-restricted conditions.

### Iron-dependent and -independent essential genes

**Fig. 3:**
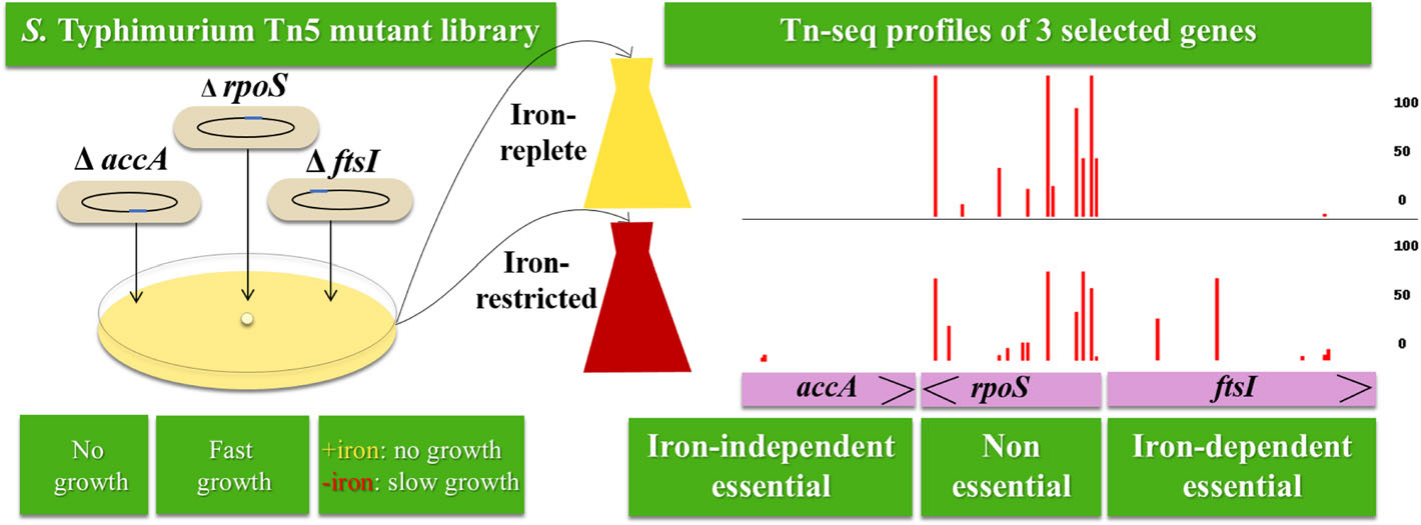
Illustration of the iron-independent essential and iron-dependent essential genes as identified by Tn-seq. A genome-saturating library *S.* Typhimurium Tn5 mutants was inoculated to iron-replete (LB medium) and iron-restricted conditions (LB medium supplemented with an iron chelator, 2,2`-Dipyridyl). The cultures were grown till mid-log phase and then subjected to Tn-seq to identify essential genes under each of the two conditions. Examples of iron-independent essential gene (*accA*), non-essential gene (*rpoS*) and iron-dependent essential gene (*ftsI*) are shown. For *accA* gene, no insertion mutant was recovered from both conditions. For *ftsI* gene, on the contrary, insertion mutants were recovered in iron-restricted condition, but not in iron-replete condition

We came to the conclusion that iron-restriction alleviated the immediate killing or growth arrest of the mutants in the 121 iron-dependent essential genes, allowing growth of the mutants, although the underlying mechanism(s) is unknown. Here, we propose to classify the essential genes in *S.* Typhimurium conceptually into two categories according to their dependency of the gene essentiality on iron concentration: iron-dependent and iron-independent essential genes (Fig. 1). The 121 essential genes that allowed growth of the mutants under iron-restricted conditions are iron-dependent essential genes (Figs. 1 and 3, and Additional file 1: Table S6). On the contrary, the 215 essential genes that did not tolerate growth of the mutants regardless of iron concentration are iron-independent essential genes (Figs. 1 and 3, and Additional file 1: Table S5).

One note for caution for this classification is that for any iron-independent essential genes reported in this study the classification can be changed potentially. It is because the decision on gene essentiality using Tn-seq data depends on the saturation level of the transposon library and the sequencing depth, and a substantial increase in the sequencing depth may reclassify some of the iron-independent genes as iron-dependent essential genes.

To further validate our classification, we compared the average read counts between iron-independent and iron-dependent essential genes under iron-replete (LB medium; LB-III) or iron-restricted condition (LB medium supplemented with Dip; Dip400). In LB-III, the average read counts for the 215 iron-independent and the 121 iron-dependent essential genes were similar (2.2 and 4.3, respectively). On the contrary, in Dip400 the average read counts were 9.6 and 67.9 for the same sets of 215 iron-independent and 121 iron-dependent essential genes, respectively (Additional file 1: Tables S5 and S6).

**Fig. 4:**
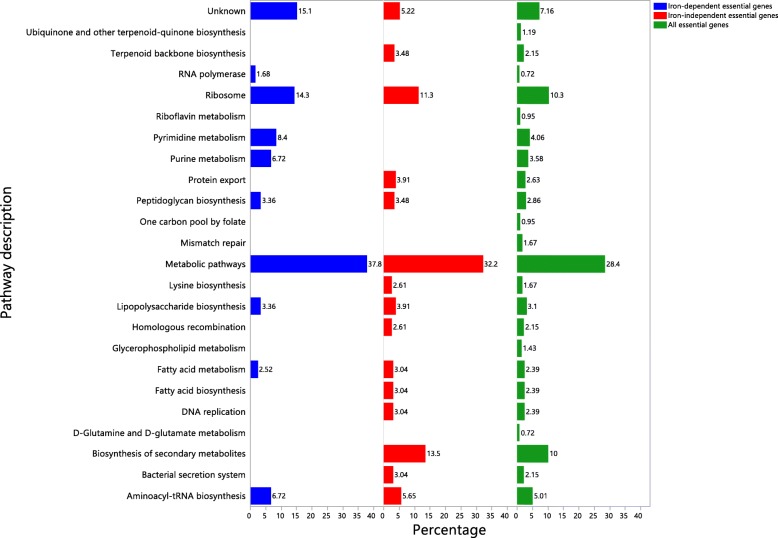
KEGG pathway enrichment analysis for the 215 iron-independent vs. 121 iron-dependent essential genes in *S.* Typhimurium 14028

In addition, we asked if the 121 iron-dependent essential genes represent unique pathways in comparison to iron-independent essential genes. The result of KEGG pathway analysis showed that iron-independent and dependent genes are represented in 15 and 10 pathways, respectively, with 7 overlapping ones (Fig. 4).

It is also important to note that all known targets of antibiotics, with only one exception of colistin, are encoded by iron-dependent essential genes. In case of *gyrA* and *gyrB*, which encode the targets of fluoroquinolones, the mutants in these genes showed significantly increased fitness under iron-restricted conditions, although they were not classified as iron-dependent essential genes (Fig. 1). It is uncertain currently if this strong bias toward iron-dependent genes reflects any mechanistic reasons underlying the preference of the antibiotic-producing microorganisms in nature to target the essential functions encoded by these essential genes. If that’s the case, it would be very intriguing to explore the possible reasons for the bias in respect to the roles of antibiotic production in their natural ecological environments.

## Discussion

It has been well established that iron contributes to cell death due to various causes**.** 2,2`-Dipyridyl iron chelator inhibits the Fenton reaction, which is required for ROS generation [5, 20]. We speculate that following disruption of the essential genes with transposon insertions, ROS production might contribute to the death or growth arrest of the mutants depending on the target genes, in addition to the disruption of the essential protein functions. We reason that our results have significant implications in understanding and expanding the current model of ROS-mediated common killing mechanisms of bactericidal antibiotics. Since its first proposal by Kohanski et al. [5], this hypothesis has been substantiated by numerous studies using different bacterial species and bactericidal antibiotics [21–24]. Traditionally, the mechanisms of antibiotic action have been studied largely in terms of antibiotic-target interactions. However, numerous researches supporting the ROS-mediated killing mechanism have shown that the interaction of antibiotic-target leads to production of ROS, contributing to the killing activity mediated by direct blocking of the essential pathways in the bacterial cells. In our study, we did not use bactericidal antibiotics to disrupt an essential pathway. However, inactivation of the essential gene functions was achieved in a permanent and irreversible manner through Tn5 insertions in the genes, instead of reversible antibiotics treatment. In most studies focused on understanding the ROS-mediated killing mechanisms of bactericidal antibiotics, the experiments were conducted using optimized sub-lethal concentrations of relevant antibiotics to allow reliable measurements of the quantitative changes in killing effects (e.g. by addition of iron chelator) [5]. However, inactivation of the essential protein functions by Tn5 insertions in our study is expected to exhibit the lethal effect equivalent to or even stronger than that caused by high concentrations of bactericidal antibiotics. This situation may limit the effect of iron chelation to very small, making it difficult to detect and measure reduction in killing caused by iron chelator. However, the Tn-seq procedure conducted with deep sequencing in combination with the high concentrations of Dip (up to 400 μM) employed in this study allowed to detect the small reductions in killing or growth arrest under the iron-restricted conditions.

Until now the ROS-mediated killing mechanism has been studied and discussed in the context of a few selected bactericidal antibiotics and their molecular targets. Our Tn-seq data show that this ROS-mediated killing mechanism might be linked to at least one-third of the essential genes in *S.* Typhimurium, thereby potentially expanding the ROS-mediated lethal pathway as a more general mechanism connected to a broad range of basic essential pathways in *S.* Typhimurium.

We believe that our findings have profound implications for understanding current crisis in public health due to the rapid increase in antibiotic resistance to most antibiotics in clinical use. Our deeper understanding of the underlying mechanisms would help in development of novel antibiotics with inherent mechanisms for reduced chance of developing drug resistance. Here, we propose that iron-independent essential genes may serve as better targets for antibiotic development for the following reasons: it has been shown that there are two opposing aspects of ROS-mediated killing mechanism. When ROS production is high due to high concentrations of bactericidal antibiotics, it would lead to facilitated killing of bacterial cells. On the contrary, when ROS is produced at low levels by sub-lethal concentrations of bactericidal antibiotics, it would lead to production of drug-resistant mutants through mutagenic action of ROS on DNA [25]. When *Salmonella* infects the host, iron-restricted host niches would suppress ROS production from bactericidal antibiotics, which would in turn reduce ROS-mediated killing process and thus overall killing effect by the antibiotics. Furthermore, depending on the iron restriction levels, reduced local concentrations of antibiotics in the host tissues might facilitate production of low levels of ROS, contributing to the production of antibiotic resistant mutants through its mutagenic action of ROS on DNA. In contrast, if a certain antibiotic targets an iron-independent essential pathway (such as those encoded by the 215 iron-independent essential genes discovered in this study), we speculate that since ROS production is not a part of their lethal processes, blocking the pathways will lead to iron concentration-independent killing, without increasing the chance of developing antibiotic resistant population via mutagenic action of ROS [25]. Our Tn-seq results show clearly that mutants of iron-dependent essential genes can grow slowly in iron-restricted conditions, and the same phenomenon may occur in the host, because iron-restriction by host is a vital mechanism to combat pathogens. As a result, it may be hard to completely kill, and eliminate *Salmonella* by blocking iron-dependent essential genes. Conversely, blocking iron-independent essential pathways would allow immediate killing of *Salmonella* regardless of iron concentration. Thus, the possibility will be higher to eradicate this pathogenic bacterium by targeting the iron-independent essential pathways in comparison to iron-dependent essential pathways as is the case for all antibiotics in clinical use except for colistin.

One common mechanism that bacteria exploit for development of antibiotic resistance is alteration of drug interaction site. Our results emphasize that the majority of genes encoding currently known drug targets are iron-dependent essential genes (Fig. 1). Prevalence of antibiotic resistance in clinical isolates due to mutations in drug targets has been rising. Mutations in a peptidoglycan synthesis gene *fts* which is the target of β-lactams in *Haemophilus influenzae* cause resistance to antibiotics [26, 27]. *E. coli* strains harboring mutations in *murA* are resistant to Fosfomycin [28]. UDP-N-acetylglucosamine enolpyruvyl transferase (MurA) catalyzes the reaction in the first step of biosynthesis of peptidoglycan in bacterial cell wall, and the protein is the target of fosfomycin [29]. Our Tn-seq results show that *murA* mutants did grow very well in iron-restricted conditions and the mutants had 14,981 read counts in Dip400 but there were only 5 reads of these mutants in LB-III (Fig. 2). It has been reported that *Pseudomonas putida* develops intrinsic fosfomycin resistance due to the presence of a salvage pathway that bypasses de novo biosynthesis of MurA [30]. Since *murA* is an iron-dependent essential gene in our study, we reason that almost all *murA* mutants died effectively in LB-III because of the contribution of ROS in the death process. However, in Dip400, reduced ROS formation and the salvage pathway biosynthesis of MurA might have caused *S*. Typhimurium to multiply and grow. Further, Fluoroquinolone-resistant bacteria are also present in clinical isolates due to mutations in drug targets, *gyrA*, *gyrB*, *parC*, and *parE*, in pathogens such as *Shigella flexneri* [31], *Salmonella* Typhi [32], and group B *Streptococcus* [33]. Rifampin-resistant *Mycobacterium tuberculosis* isolates are associated with mutations in their targets, *rpoB* and *rpoC* [34, 35]. Mutations in *rplC* contributed to *Staphylococcus aureus* resistance to linezolid in a clinical isolate [36]. Finally, Telithromycin resistant-isolates of *S. aureus* due mutations in a ribosomal gene, *rplB*, were detected in vitro [37]. All together, these antibiotic targets, which are the products of iron-dependent essential genes, can mutate the target genes and alter the structure of corresponding proteins in order to evade lethal interactions with the antibiotics.

One example of an antibiotic targeting a protein encoded by iron-independent essential gene is colistin. Colistin (polymyxin E) is a last resort antibiotic for treatment of infections caused by multidrug resistant Gram-negative bacteria [38]. This bactericidal drug interacts with the lipid A moiety of lipopolysaccharide (LPS) and ultimately causes membrane lysis [39]. We showed that the genes encoding the molecular targets of Colistin, *lpxABCDHK* are iron-independent essential genes. Over the last 60 years, Colistin has been used for fighting infectious diseases, but with caution and limitation due to its known toxicity. This resulted in less frequent use of Colistin, which has been considered as the main reason why drug resistance is low for Colistin. Our Tn-seq results indicate that disruption of LPS is lethal in *S*. Typhimurium and there is no contribution of ROS to death process caused by Colistin. Supporting our finding, a study found that killing of *Pseudomonas aeruginosa* by Colistin is ROS-independent and ROS scavengers does not reduce the killing process [40]. However, another study demonstrated that Colistin-induced killing in *Acinetobacter baumannii* involves ROS production [41]. These contradicted findings are not surprising, considering that there have been continued debates on the common antibiotic killing mechanism via ROS. Although this model is widely accepted, a few studies have challenged it [42, 43].

## Conclusion

**Fig. 5:**
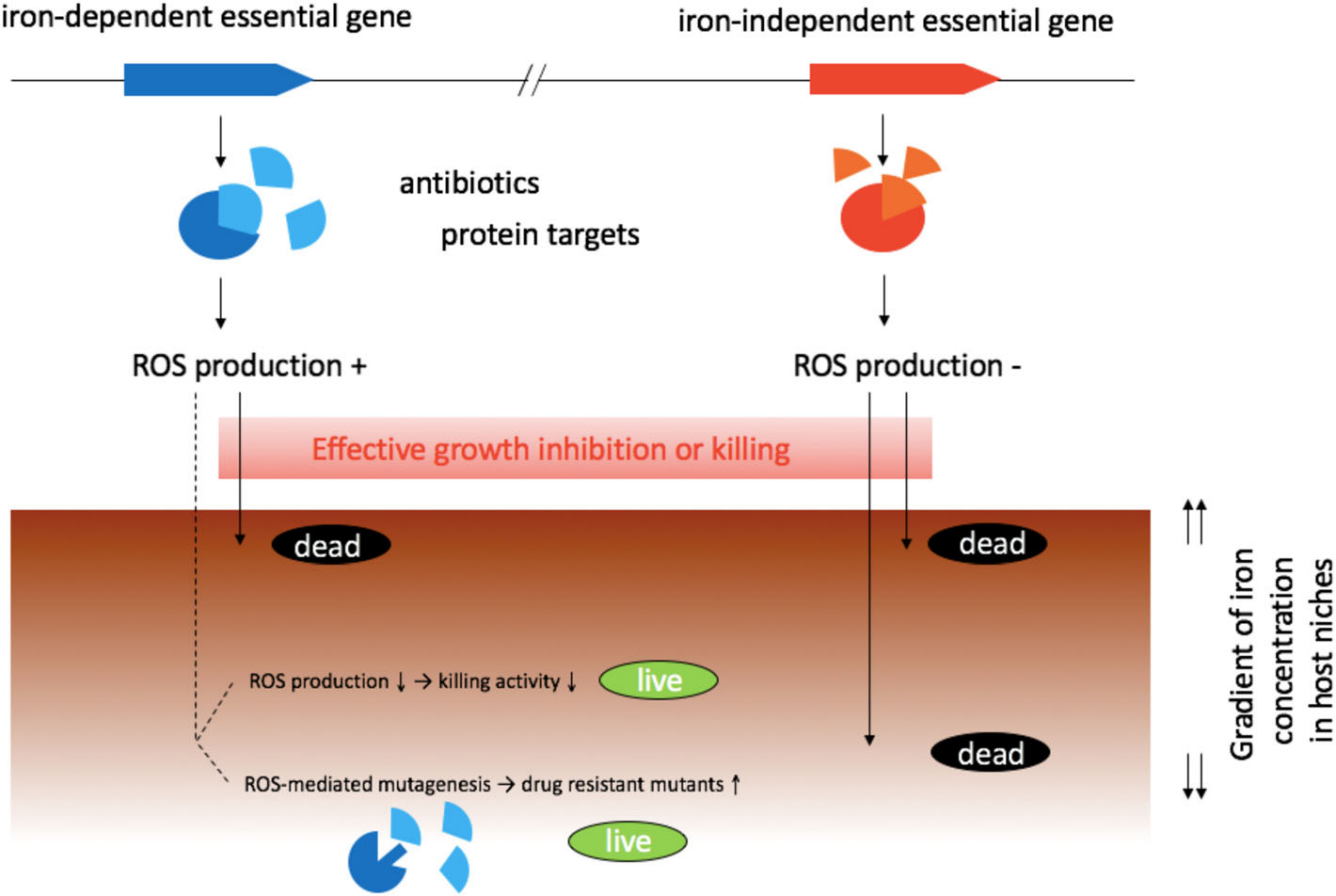
Current models for iron-dependent vs. iron-independent essential genes, in which the production of reactive oxygen species (ROS) is a critical component separating all essential genes into these two categories. When the protein function encoded by an iron-dependent essential gene is blocked, ROS is produced, contributing effective killing of bacterial cells in iron-replete conditions. However, ROS production would decrease in iron-restricted conditions (e.g. host tissues) reducing the killing activity and facilitating production of drug resistance bacteria via mutagenic action of sub-lethal concentration of ROS. On the contrary, blocking the protein function encoded by an iron-independent essential gene would lead to effective bacterial killing, regardless of the iron concentrations

In this work we employed Tn-seq to elucidate the genes exhibiting iron-dependent or iron-independent essential phenotypes in *S.* Typhimurium when inactivated. Our Tn-seq data indicated that when transposon mutant library was treated with an iron chelator, the mutants of approximately one-third of previously known essential genes escaped immediate killing or growth inhibition, and multiplied slowly, increasing their relative abundance. Based on this observation, we speculate that the iron chelator reduces ROS formation via inhibition of the Fenton reaction, thereby alleviating the lethal outcomes following inactivation of the essential genes. Accordingly, we classified 336 essential genes in *S.* Typhimurium 14028 into iron-independent vs. iron-dependent essential genes depending on their dependency of the essentiality on iron concentration. We propose that iron-independent essential genes and their proteins may serve as better targets to develop new antibiotics, because targeting these pathways would lead to immediate killing or growth inhibition, regardless of the local concentrations of iron in the host milieu. The proposed model illustrating our concept of iron-dependent and iron-independent essential genes is summarized in Fig. 5. Obviously, further studies providing experimental evidences on the direct involvement of ROS production in the process would be necessary to fully upport our model. Due to the unique difficulty associated with studying essential genes and their mutants, the use of inducible knockouts such as CRISPRi [17] would be helpful in elucidating the role of ROS in the process following inactivation of the essential genes. It would be also interesting to see if we could come to the similar conclusion using the same Tn-seq approach, when thiourea, hydroxyl radical scavenger, is used in place of Dip to suppress ROS production or the mutants are cultured anaerobically.

This study provides new insights on the previously unknown aspect of the essential genes and the essential pathways or functions encoded by them in *Salmonella.* Although further studies are needed to gain better understanding on the scope of this observation (e.g. other bacteria), and to elucidate mechanistic basis of the iron-dependent essential genes, this study points us to a new direction of research that would be important to understand and overcome current crisis of antibiotic resistance.

## Methods

### Bacterial strains and Tn5 mutant library construction

*Salmonella* enterica subsp. enterica serovar Typhimurium ATCC 14028 with spontaneous mutation conferring resistance to nalidixic acid (NA) was used in this study. All procedures involving this pathogen (Biosafety level 2) were conducted according to the protocol approved by Institutional Biosafety Committee (IBC) at the University of Arkansas. Transposon mutant libraries were prepared as previously described [18]. Briefly, *S.* Typhimurium ATCC 14028 NA^R^ (the recipient strain) was subjected to transposon mutagenesis by biparental mating using *Escherichia coli* SM10 λ*pir* carrying a pBAM1 transposon-delivery plasmid vector [44] as the donor strain. An equal volume of the overnight growth cultures of the donor and recipient strains were washed with 10 mM MgSO_4_ and concentrated on the nitrocellulose filter, which was then incubated for 5 h at 37 °C on a surface of LB agar plate. After the incubation, the cells were washed with 10 mM MgSO_4_ and plated on LB agar plates containing 50 μg/ml NA and 50 μg/ml Kanamycin (Km). The plates were incubated at 37 °C for 24 h. Then, LB broth supplemented with 7% DMSO was added onto the plates, and the colonies were scrapped off, resuspended, and stored at − 80 °C in aliquots. We constructed two mutant libraries, A and B. Each library contains approximately 325,000 mutants. The numbers of mutants in the libraries were estimated based on the average number of colonies recovered from each conjugation.

### Growth rate measurement for *S.* Typhimurium

A single colony of *S.* Typhimurium ATCC 14028 was inoculated into 2 ml LB broth medium in a 5 ml tube and incubated overnight (~ 16 h). Freshly prepared LB broth media supplemented with different concentrations of 2,2`-Dipyridyl **(**Dip) were inoculated with the overnight culture at a 1:200 dilution. The cultures were immediately added into the wells of a 96-well microplate (200 μl/well) and incubated in a TECAN Infinite 200 microplate reader at 37 °C (shaking amplitude of 1.5 mm, and shaking duration of 5 s). OD_600_ was measured every 10 min for 24 h. The collected data were used to determine lag time, growth rate, and maximum OD_600_ for each concentration using GrowthRates script [45].

### Selection of mutant libraries for Tn-seq analysis

An aliquot of the transposon libraries in stock was thawed at room temperature and diluted 1:10 in LB broth. The library was incubated at 37 °C with shaking at 225 rpm for an hour and then washed twice in PBS. The activated culture of Library-A was inoculated to 20 ml LB broth (LB-II) and LB broth supplemented with either 100 (Dip100) or 150 μM Dip (Dip150) in a 300 ml flask, with seeding CFUs of 3.5 × 10^6^ per ml. We also included LB-I for which Library-A was directly subjected to Tn-seq after activation and washing. In addition, to accomplish a higher saturation level, Library-A was combined with Library-B (termed Library-AB; Additional file 2: Figure S1). The activated culture of Library-AB was inoculated to 20 ml LB broth (LB-III) and LB broth supplemented with either 250 (Dip250-I and Dip250-II) or 400 μM Dip (Dip400) in a 300 ml flask, with seeding CFUs of 8 × 10^6^ per ml. Dip100, Dip150, Dip250-I, Dip250-II, and Dip400 as well as LB-II and LB-III were incubated at 37 °C with shaking at 225 rpm until the cultures reached mid-log phase (OD_600_ of ~ 2.7). Then, the cultures were immediately centrifuged to get the cell pellets, which were stored at − 20 °C for the downstream analysis.

### Preparation of Tn-seq libraries for HiSeq sequencing

The preparation of Tn-seq libraries was performed as previously described [18]. Briefly, genomic DNA was extracted from each sample using DNeasy Blood & Tissue kit (Qiagen), and quantified using Qubit dsDNA RB Assay kit (Invitrogen). To remove the pseudo Tn5 mutants generated by chromosomal integration of pBAM1, genomic DNA was digested with PvuII-HF (New England Biolabs), and purified with DNA Clean & Concentrator-5 kit (Zymo Research). Then, a linear PCR extension was performed using Tn5-DPO (5’-AAGCTTGCATGCCTGCAGGTIIIIICTAGAGGATC-3′). The PCR reaction was performed in a 50 μl reaction containing GoTaq Colorless Master Mix (Promega), 20 μM Tn5-DPO primer, 100 ng gDNA, and H_2_O. The PCR cycles consisted of 95°C for 2 min, followed by 50 cycles at 95°C for 30 s, 62°C for 45 s, and 72°C for 10 s. The PCR product was purified with the DNA Clean & Concentrator-5 kit. The C-tailing reaction was conducted with terminal transferase (TdT; New England Biolabs), CoCl_2_, dCTP, ddCTP, TdT buffer, and the purified linear PCR product. dCTP and ddCTP were included at the molar ratio of 20:1. The mixture was incubated at 37°C for 1 h, which was followed by 10 min incubation at 70°C for inactivation of TdT. The C-tailed product was purified. Next, the exponential PCR was performed with P5-BRX-TN5-MEO primer, AATGATACGGCGACCACCGAGATCTACACTCTTTCCCTACACGACGCTCTTCCGATCTNNNNAG-BC-CCTAGGCGGCCTTAATTAAAGATGTGTATAAGAG (where “BC” denotes different sample index barcodes of 8 nt long) and P7-16G primer, CAAGCAGAAGACGGCATACGAGCTCTTCCGATCTGGGGGGGGGGGGGGGG. The PCR reaction was performed in a 50 μl reaction containing GoTaq Green Master Mix, P5-BRX-TN5-MEO primer, P7-16G primer, purified C-tailed Tn5-junction fragments, and H_2_O; the PCR cycles consisted of 95°C for 2 min, followed by 30 cycles of 95°C for 30 s, 60°C for 30 s, and 72°C for 20 s, with the final extension at 72°C for 5 min. Then, the 50 μl PCR products were separated on an agarose gel, and the DNA fragments of size 325–625 bp were extracted using Zymoclean Gel DNA Recovery kit (Zymo Research). The DNA libraries were quantified using Qubit dsDNA RB Assay kit. The DNA libraries were combined and sequenced on a flow cell of HiSeq 3000 using single-end read option for 151 cycles at the Center for Genome Research & Biocomputing in Oregon State University.

### Analysis of Tn-seq data

The HiSeq sequence results were downloaded onto High Performance Computing Center (AHPCC) at the University of Arkansas. The sequence reads were de-multiplexed using a custom Python script. The script searched for the unique 8-nucleotide index barcode of each library for perfect matches. The transposon genomic junctions were extracted using Tn-Seq Pre-Processor (TPP) tool [46]. The TPP searched for the 19 nucleotide inverted repeat (IR) and identified five nucleotides (GACAG) at the end of the IR sequence, allowing one nucleotide mismatch. The genomic junctions that start immediately after the GACAG were extracted and the C-tails were removed. The junction sequences of less than 20 nucleotides were removed and remaining junction sequences were mapped to the *S.* Typhimurium 14028 genome and plasmid using BWA-0.7.12 [47]. The TPP counted and reported the number of total sequence reads after filtering, total mapped read, and total unique insertions in each library.

### Identification of essential genes

LB-I, LB-II, and LB-III were analyzed to identify the essential genes in *S*. Typhimurium 14028 in LB medium. We used two different tools for Tn-seq essential gene analysis. First, TRANSIT [46] analysis of essentiality on gaps in entire genome was conducted using tn5gaps algorithm. Only the insertions inside the 5% of N-terminal and 10% of C-terminal ends of all open reading frames (ORF) were included and even insertions with only one reads were considered for the analysis. The gene was considered essential if its *p* value ≤0.05. Secondly, Tn-Seq Explorer [48] was used for essential gene analysis by applying a 550 window size. The insertions in the 5% of N-terminal and 10% of C-terminal ends of all ORFs were removed, and insertions with only one reads were included in the analysis. The gene was considered essential if its Essentiality Index (EI) was ≤2. Then, the essentiality analysis results by both methods were combined. Finally, we demanded that for a gene to be considered essential for growth on LB medium (both LB agar and LB broth) the following three criteria should be met: (i) the gene is essential in LB-III by both tools, Tn-Seq Explorer and TRANSIT, and (ii) the gene is essential in at least 5 out of the 6 analyses that were performed for the LB-I, LB-II, and LB-III by the two analysis tools (Additional file 2: Figure S4). We made exceptions for the 17 genes for which the requirements for essential genes in at least 5 out of the 6 analyses were reduced to 4 out of 6. These exceptions were based on the observations that these 17 genes were previously identified as essential genes both in *S.* Typhimurium SL326 [13] and *E. coli* K12 [10, 11] and slightly missed the threshold for calling essential genes in this study. The list of all essential genes we identified according to our stringent conditions is shown in Additional file 1: Table S3. The same strategy was used to identify essential genes under iron-restricted conditions (Dip250-I, Dip250-II, and Dip400).

### Calculation of fitness for selected essential genes

Mutant fitness under iron-restricted conditions for the iron-dependent essential genes was analyzed by using TRANSIT, with resampling option. LB-I was used as the input for Dip100 and Dip150, while LB-III was used as the input for Dip250-I, Dip250-II, and Dip400. For data normalization for fitness calculation, Trimmed Total Reads (TTR) option in TRANSIT was used and 10,000 samples were used for the analysis. The insertions in the 5% of N-terminal and 10% of C-terminal ends of ORFs were removed and the gene fitness was considered significant if *p* value was ≤0.05.

## Additional files

Additional file 1: **Table S1.** Summary of the Tn5 mutant libraries used for inoculation and recovered from selections. **Table S2.** Summary of the Tn5-junction sequence reads in this study. **Table S3.** Complete list of *S.* Typhimurium 14,028 essential genes in iron-replete condition (LB medium) identified by Tn-seq. **Table S4.** The essential genes commonly identified between this study (*S.* Typhimurium 14,028 in LB medium) and the previous study (*S*. Typhimurium SL3261) using TraDIS. **Table S5.** The iron-independent essential genes in *S.* Typhimurium 14,028 identified in this study. **Table S6.** The iron-dependent essential genes in *S.* Typhimurium 14,028 identified in this study. **Table S7.** The average sequencing read counts in the essential genes of *S.* Typhimurium 14,028 in iron-replete (LB) and iron-restricted conditions (Dip400) from Tn-seq data. **Table S8.** The iron-dependent essential genes identified in this study are also essential genes in other bacteria. **Table S9.** Summary of the sequencing read counts in all ORFs in iron-replete (LB-III) vs. iron-restricted conditions (Dip250-I, Dip250-II, and Dip400). (XLSX 138 kb)

Additional file 2: **Figure S1.** Schematic representation of the study design. **Figure S2.** Effect of 2,2`-Dipyridyl (Dip) on *S.* Typhimurium growth. **Figure S3.** Effect of 2,2`-Dipyridyl (Dip) on *S.* Typhimurium growth rate and cell density. **Figure S4.** Algorithm used for essential gene calling. **Figure S5.** KEGG pathway analysis of the 336 essential genes of *S.* Typhimurium 14,028 in LB medium identified in this study. (PDF 608 kb)

## Abbreviations

CRISPRi: Clustered Regularly Interspaced Short Palindromic Repeats Interference
Dip: 2,2`-Dipyridyl
ROS: Reactive oxygen species
Tn-seq: Transposon sequencing
