# Supplementary material for: Iron-dependent essential genes in Salmonella Typhimurium
Source: BMC Genomics. 2018 Aug 14;19:610. doi: 10.1186/s12864-018-4986-1 (PMC6092869; doi:10.1186/s12864-018-4986-1)
Supplement: Supplementary file 2 — Figure S1. Schematic representation of the study design. Figure S2. Effect of 2,2`-Dipyridyl (Dip) on S. Typhimurium growth. Figure S3. Effect of 2,2`-Dipyridyl (Dip) on S. Typhimurium growth rate and cell density. Figure S4. Algorithm used for essential gene calling. Figure S5. KEGG pathway analysis of the 336 essential genes of S. Typhimurium 14,028 in LB medium identified in this study. (PDF 608 kb) [file 12864_2018_4986_MOESM2_ESM.pdf]

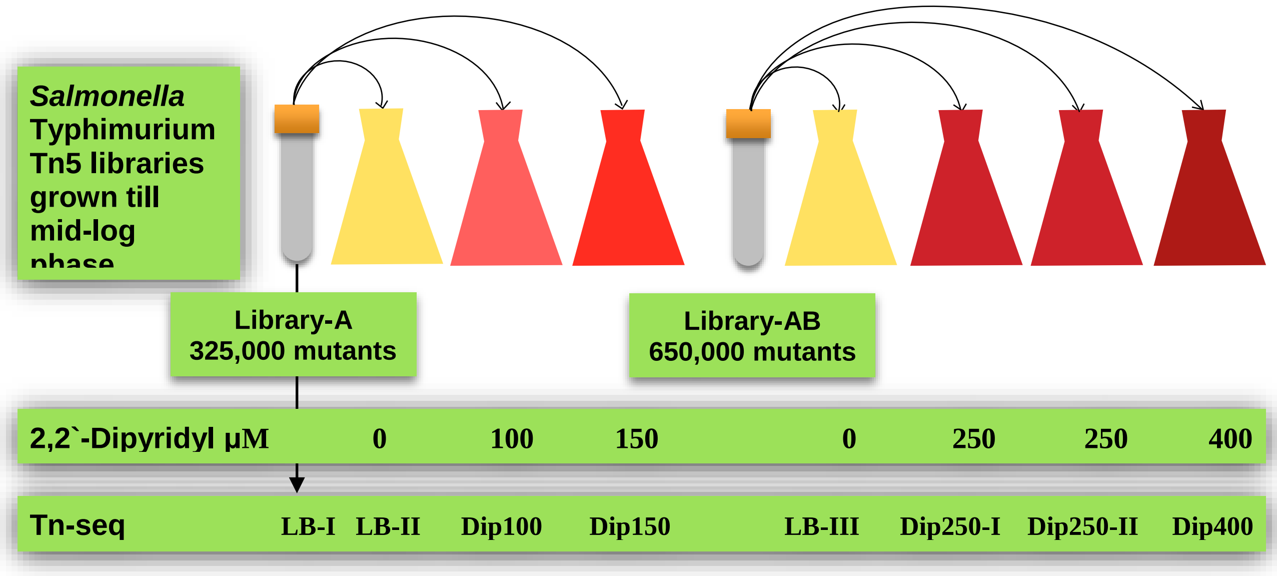

Figure S1. Schematic representation of the study design. Library-A was inoculated to LB medium (LB-II) or LB medium containing either 100 µM iron chelator Dip (Dip100) or 150 µM Dip (Dip150). Library-A was also directly subjected to Tn-seq analysis without growth (LB-I). Library-AB was inoculated to LB medium (LB-III) or LB medium containing either 250 µM Dip (Dip250) or 400 µM Dip (Dip400). The cultures were grown till mid-log phase and then subjected to Tn-seq analysis.

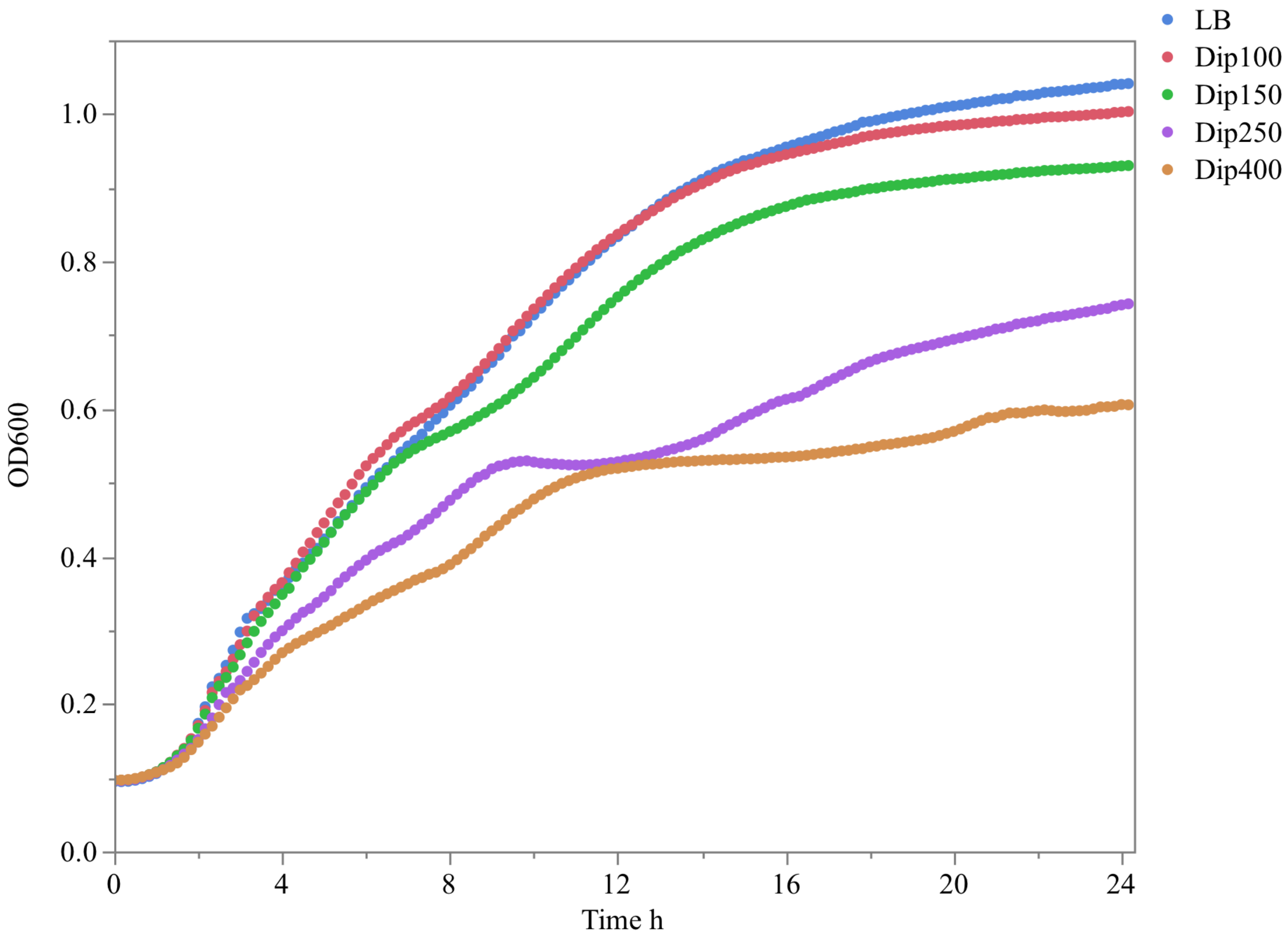

Figure S2. Effect of 2,2`-Dipyridyl (Dip) on *S.* Typhimurium growth. An overnight culture of the wild type *S.* Typhimurium 14028 was diluted 1:200 in LB medium supplemented with 0 µM Dip (LB), 100 µM Dip (Dip150), 150 µM Dip (Dip150), 250 µM Dip (Dip250) or 400 µM Dip (Dip400). The cultures were transferred to the wells of a 96-well plate and incubated at 37˚C in a plate reader with reading OD_{600} every 10 minutes.

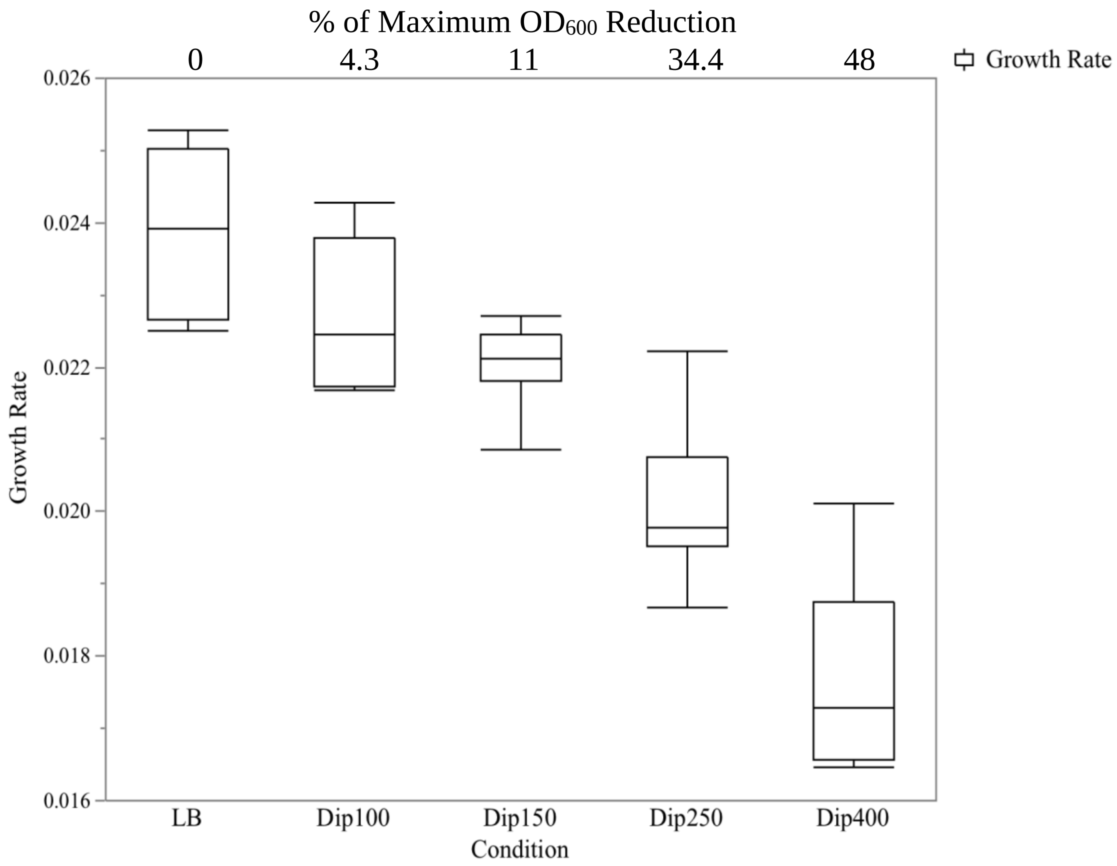

Figure S3. Effect of 2,2`-Dipyridyl (Dip) on *S.* Typhimurium growth rate and cell density. An overnight culture of the wild type *S.* Typhimurium 14028 was diluted 1:200 in LB medium supplemented with 0 μM Dip (LB), 100 μM Dip (Dip150), 150 μM Dip (Dip150), 250 μM Dip (Dip250) or 400 μM Dip (Dip400). The cultures were transferred to the wells of a 96-well plate and incubated at 37°C in a plate reader with reading OD_{600} every 10 minutes. The maximum OD_{600} reduction is shown as a percentage in reference to LB.

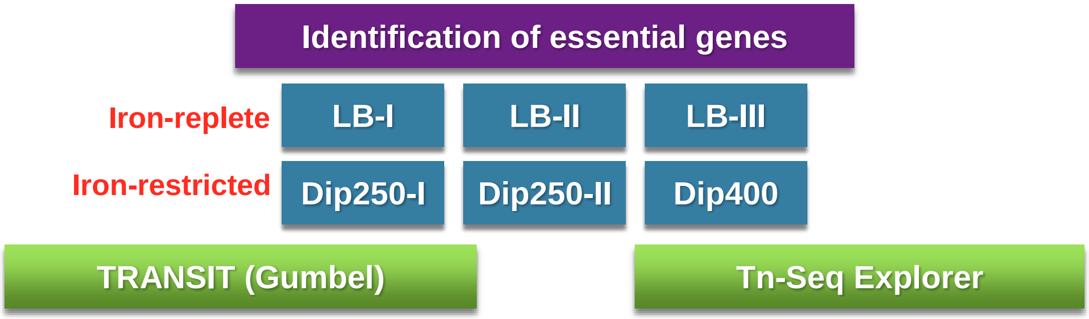

**A**		LB-III Essentiality Index < 3
**B**	LB-III Essential p < 0.05	
**C**	The gene has to be essential in at least 5 of the 6 essentiality analyses	

Essential genes in iron-replete condition

**Gene**	**LB-I**	**LB-II**	**LB-III**		**LB-I**	**LB-II**	**LB-III**	**Call**
murA	**E**	**E**	**E**		**0**	**0**	**0**	**E**
dapD	**NE**	**E**	**E**		**1**	**1**	**0**	**E**
acpP	**NE**	**NE**	**E**		**2**	**1**	**1**	**NE**
gmhA	**NE**	**NE**	**E**		**5**	**1**	**0**	**NE**
ssb	**E**	**NE**	**E**		**3**	**0**	**1**	**NE**
cydC	**E**	**E**	**E**		**3**	**0**	**1**	**E**

Essential genes in iron-restricted condition

**Gene**	**Dip250-I**	**Dip250-II**	**Dip400**		**Dip250-I**	**Dip250-II**	**Dip400**	**Call**
murA	**NE**	**E**	**E**		**4**	**2**	**4**	**NE**
dapD	**E**	**NE**	**E**		**0**	**2**	**2**	**E**
acpP	**NE**	**E**	**NE**		**2**	**1**	**2**	**NE**
gmhA	**NE**	**E**	**NE**		**1**	**0**	**1**	**NE**
ssb	**NE**	**NE**	**E**		**4**	**2**	**3**	**NE**
cydC	**E**	**E**	**E**		**2**	**4**	**5**	**NE**

Figure S4. Algorithm used for essential gene calling. Two methods were used for essential gene analysis, TRANSIT (Gumbel) and Tn-Seq Explorer. Tn-seq data from LB-I, LB-II, and LB-III were analyzed separately by both methods for identification of essential genes in iron-replete condition. A gene was considered essential if 5 out of 6 analyses (3 groups x 2 methods) were essential (E; TRANSIT) or essentiality index (EI; Tn-Seq Explore) < 3. The essential genes in iron-restricted condition were identified using the same algorithm but with Tn-seq data from Dip250-I, Dip250-II, and Dip400.

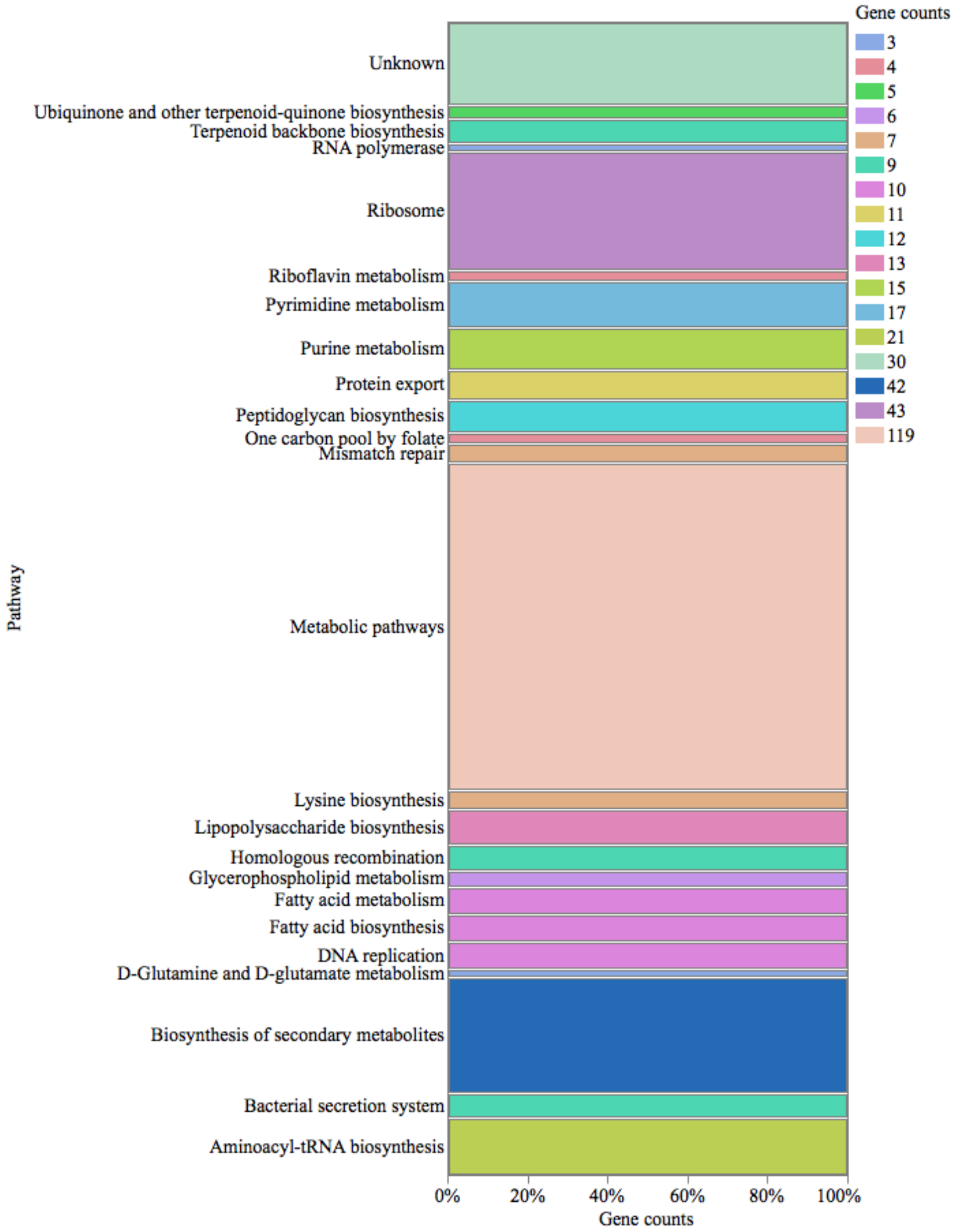

Figure S5. KEGG pathway analysis of the 336 essential genes of *S.* Typhimurium 14028 in LB medium identified in this study. All 336 essential genes were categorized into 23 essential KEGG pathways.
